# More Than Their Bodies. Aesthetic Dimensions of Nursing Care to People With Eating Disorders

**DOI:** 10.1111/scs.70270

**Published:** 2026-06-04

**Authors:** Sine Maria Herholdt‐Lomholdt, Berit Støre Brinchmann

**Affiliations:** ^1^ Faculty of Nursing and Health Sciences Nord University Bodø Norway; ^2^ Research Group: Caring in Healthcare, Faculty of Nursing and Health Sciences Nord University Bodø Norway; ^3^ Regional Centre for Eating Disorders Nordland Hospital Trust Bodø Norway

**Keywords:** aesthetic, care, eating disorder, nursing care, psychiatric nursing

## Abstract

**Aims and Objectives:**

This study aims to explore what it means and what it takes to focus on ‘more than the body’ in nursing care for hospitalized people with an eating disorder.

**Methodological and Justification:**

Eating disorders are complex mental disorders with a high mortality. Nursing care in this context usually focuses on mealtime activities and nutritional support to help patients survive. Nevertheless, earlier studies from a patient perspective call for nursing care, focusing on more than the body and the patient's physical survival. The study is designed phenomenologically and is theoretically informed by philosophic aesthetics by the Danish philosopher, Dorthe Jørgensen.

**Ethical Issues and Approvals:**

The study is approved by the Norwegian Data Protection service, SIKT (ref. number 947253).

**Research Methods:**

The study is based on a phenomenological analysis of a single lived experience description told by an experienced nurse working within the field of eating disorders.

**Results:**

The analysis shows that focusing on ‘more than the body’ implies invitations to people with eating disorders to participate in everyday activities which they earlier enjoyed. This means invitations into experiences of meaning and liveliness.

**Study Limitations:**

The study is based on one lived experience description expressed by an experienced nurse. This is commonly accepted in phenomenological research as the results point to possible human experiences of importance. Nevertheless, this is also a limitation, as other stories could point elsewhere and as the patient's own perspectives are not included.

**Conclusion:**

The study recommends nurses to be courageous enough to include activities that let the patient experience meaning and liveliness in the care of people with severe eating disorders.

## 1 Introduction

Eating disorders (EDs) are complex mental disorders associated with serious physical and psychological consequences and a high mortality rate [1, 2, 3]. Nursing care for patients with serious EDs usually focuses on mealtime activities and nutritional support [4, 5, 6] as well as physical health monitoring and feeding [6, 7]. This leads to care primarily focusing on remission of the disease.

However, de Vos et al. [8] show that patients with an ED experience other dimensions than remission of the ED as important to recovery. In their qualitative meta‐analysis on recovered patients' own perspectives, psychological well‐being was found to be an indicator of ED recovery. de Vos et al. [8] show a strong connection between recovery, having positive relations with others and a feeling of life having purpose and meaning. Based on this review, de Vos et al. [8] recommend that the treatment and care of patients with EDs also include positive aspects of health. The findings of de Vos et al. [8] correspond to findings of Nilsen et al. [9], who show that some young persons with anorexia nervosa (AN) conceptualize the ED as a coping strategy and that most of the young persons included in the study emphasized the necessity of letting go of the ED or ED behaviours to recover. Building relationships around better things than just thinking of food and focusing on things that really matter are described as crucial for recovery [9].

Further, this perspective is underlined by Rance et al. [10] studying perspectives of adult women, who have been under treatment for AN. These women criticized the treatment regime for being ‘overly focused on food and weight’ [10] p. 586 and recounted that treatment and therapy focusing narrowly on food and weight gain ‘had the potential to encourage them to remain in, rather than relinquish, their ED’ [10] p. 587. The study of Rance et al. [10] suggests that, alternatively, genuine relationships with healthcare staff and therapists and the possibility of being seen and addressed as whole persons are much more helpful during the recovery process.

The studies of Rance et al. [10], Nilsen et al. [9] and de Vos et al. [8] call for nursing care of people with EDs, focusing on *more* than the body and the ED. This means focusing on more than feeding, mealtime activities, weight monitoring and nutritional support. However, the studies of Rance et al. [10], Nilsen et al. [9] and de Vos et al. [8] fail to visualize a wider picture of such nursing care, why this study further ask, how such care can be practiced and what it requires. This study responds to the call from patients under treatment for EDs, by posing the overall research question: *What does it mean and what does it take to focus on* ‘more than the body’ *in nursing care for people with a severe ED?*

## 2 Methods

This study is part of a larger qualitative research project named ‘EDs and care*’* [11, 12, 13]. The purpose of the overall project was to inquire into nurses’ perspectives of good nursing care of hospitalized people with an ED. Our study builds on an existential phenomenological approach as expressed and developed by van Manen [14, 15, 16, 17].

### 2.1 Data Collection

As accepted in van Manen's phenomenological research [14], data in this study consist of one lived experience description and a dialogue around this lived experience between a nurse and the second author of this article. The nurse, named Martin, had long experience of psychiatric care—specifically within the field of EDs. The lived experience description and dialogue came to presence through a phenomenological interview. As recommended in van Manen's phenomenological approach [16], the phenomenological interview focused on descriptions of lived experiences and reflections regarding these specific experiences. These lived experiences were situations where the nurse considered that the care offered did the patient well.

The interview with Martin was part of the larger research project where twelve nurses from a ward for adult patients with EDs were interviewed by the second author [11, 12, 13]. All were asked to describe one or more situations in which they had performed good nursing care. Not all the informants were equally good storytellers, and the participant chosen for this article (Martin) was the one who stood out most clearly with his long, reflective interview that made a strong impression on both authors. Martin was interviewed once. The interview was recorded, lasted 70 min, and was transcribed verbatim (66 pages).

As van Manen [18] states, phenomenology is the study of examples, and one example is enough to do a proper and insightful phenomenological study. The specific lived experience description and dialogue was chosen as an example for this study, as it addresses the call from people with an ED to focus on more than activity level and calorie intake. On that basis, we found reasons to inquire into this specific example in a phenomenological way, as an example of care focusing on ‘more than the body’.

### 2.2 Analytical Approach

The analysis is performed in three phenomenological steps inspired by van Manen's [16, 18, 19] recommendations for phenomenological research. He suggests that:

The human science researcher is not just a writer, someone who writes up the research report. Rather, the researcher is an author who writes from the midst of life experience where meaning resonates and reverberates with reflective being. Sensitive phenomenological texts reflect on life while reflecting life [16] p. 15.

Therefore, first, the lived experience description is constructed in its whole.

Second, the analysis goes through an initial reflection aiming at connections between the lived experience description and the overall research question as well as formulations of more specific analytical questions. In this phase, three questions guide the analysis: (1) *What does it mean to see* ‘more than the body’ *in nursing care for people with EDs? (2) What does it take—and how is it possible for nurses to open spaces where people with EDs also sense and take part in such ‘more’? (3) What kind of meaning and significance can such ‘more’ have for people with EDs and for the nurse offering care?*

The third step of the analysis is a phenomenological and philosophically inspired dwelling on the lived experience of seeing ‘more’ while caring for people with EDs. This part of the analysis is performed as a dialogue between the above three analytical questions, the chosen example and a philosophical aesthetic perspective.

By that, the full analytical process is documented in the result section, not just the main findings. This is where phenomenology differs as a method, as the whole process and text, loaded with evocative examples, is a central part of the findings [17].

### 2.3 Ethical Considerations

The nurses participating in the study provided informed consent. The data material was anonymised and treated confidentially. The participants were given the opportunity to read and approve the verbatim script, but no one made use of it. The study was approved by the Norwegian Data protection services for research SIKT (reference number 947253).

### 2.4 Theoretical Approach—A Philosophical Aesthetic Perspective

The philosophical aesthetic perspective used in this paper draws on the work of the Danish philosopher Jørgensen [20, 21, 22, 23] as well as her inspiration from the founder of philosophical aesthetics, Baumgarten [24]. In Jørgensen's sense [21], aesthetics is a certain kind of experience characterized by a sense of fullness and meaning. Aesthetic experiences are therefore not connected to a logical or rational form of thinking, but merely to feeling, sensation, and hunche [20, 21]. As Baumgarten [24] puts it, aesthetic experiences rely on sensitive cognition.

In Jørgensen's work [20, 21], aesthetic experiences are described as a sense of the world opening up and at the same time, a sense of belonging in this world. It can, for example, happen when we, as human beings, stand still late at night and admire a starry sky. It can also happen when we find ourselves fully absorbed in a play or a piece of music, and it can happen when a beautiful scent of flowers hits us, just to mention a few examples. This means that aesthetic experiences are not reserved for encounters with artwork, although they can also happen in such encounters [20]. Aesthetic experiences belong to life and can happen in human encounters with each other, with nature, with music, sculptures, poems, in dialogues and much more [20].

Further, Jørgensen [20, 21, 23] describes these world‐openings as experiences of immanent transcendence, meaning that from the midst of these often Iordinary experiences, a sense of something larger grows, which again is connected to a sense of deep existential meaning.

The key point here is that according to Jørgensen [20, 21, 22] everyday life can hold aesthetic experiences, and that such experiences are important for human beings as experiences of existential meaning and belonging. Moreover, aesthetic experiences can also be kept away or avoided if we as humans are present in life in an exclusively rational way.

## 3 Results

Below the three steps of the phenomenological analysis will be presented. First, we present the chosen lived experience description. Secondly, an initial reflection follows, aiming at the identification of central analytical questions. Thirdly, a phenomenological analysis and discussion of the three analytical questions are carried out. In this last part, results, reflections, and theoretically informed discussions are intertwined.

### 3.1 ‘More than my body’. A Lived Experience Description From an Experienced Nurse

We who work here are very concerned about Body Mass Index (BMI) and weight, and that is certainly important. Nevertheless, when you work here, you must consider that we as humans have other wishes, other needs, other opportunities. We, as professionals, need to have wider thoughts, focusing not solely on weight.

If the patients can be allowed to continue with activities that they love, then I find that they are sometimes torn out of the ED. Perhaps they get a bit of different input and other thoughts. Then they can have a really good time.

If you compare the ED to walking a dog on a leash, you will occasionally let the dog run. We have good contact related to playing (an instrument), or we can play a game or bingo. All such activities, that in a way can seem a bit entertaining or diverting—derailing is probably the key word here—they are very good for these patients.

Some of the patients have been here for a very long time. They might have a lot of restrictions related to going out because we don't want them to burn calories. They mostly must be by themselves and turn inwards. What should they fill all the hours with?

Most of the patients think a lot. The ED kind of dominates their ‘mindset’ very much. It is important that we can do pleasant things together. Things that interest the patient. Sometimes you must push them a little. ‘I don't really feel like it today’ ‘Yeah, come on, what are you going to do instead?’ I think I prefer to stay in my room’. ‘Okay, what are you going to do there?’ ‘Nothing’. ‘Oh, but then join us instead’. I call it an empathic push. Several times I have seen that it leads to good conversation and good times.

When we play games or play the piano or do other things, we try not to talk about illness. These people also want to be met on their healthy side.

Our patients are asked to make…… we use to call it an ‘encouragement poster’. It's a poster which they produce themselves. Afterwards this poster is in their room, and they look at it every day, to remind themselves that there is hope. Encouraging them to carry on, almost like a little life jacket.

One patient had written ‘I am more than my body’. And I asked, ‘Where did you get that quote?’ She said that she made it up herself.

At home, these patients often experience that they are seen solely as being ill by their parents and siblings. They are ashamed of experiencing themselves seen, heard, and met only as unwell. But if you can meet people in their resources, meet what they are successful at, then they get a sense of mastery. So, I would say that our job is to give patients hope and to shed some light into the dark ED. This is what I want to contribute to.

If we play a game or the piano together, we get a different kind of contact. Then, there is another project in question. My concern is that we shouldn't talk about disease and suffering all the time—twenty‐four hours a day. Instead, we can go for a walk. If they take the initiative themselves, then I'll follow up, but we can also talk about the weather and pick flowers or do something else. We can do something nice.

There is something about … it can be a bit too much. We have a lot of focus on conversation therapy. The patients have conversations with those of us who are contact nurses and with the therapists as well. There are an enormous number of conversations. I think these people also need some conversations of an easier kind. Conversations giving them slightly different inputs. Conversations focusing on other things than illness (Extract from interview with the nurse Martin).

### 3.2 Initial Reflections

Reading the above story, we found that Martin expressed two kinds of nursing for people with an ED. On the one hand Martin describes nursing care preoccupied with counting calories and therapeutic conversations, as described in the research of Hage et al. [4, 5], de Vos et al. [8], Nilsen et al. [9] and Webb et al. [6]. On the other hand, Martin talks about a nursing care preoccupied with something *more or something else* than the sick person's body. Martin realized the importance of such other focus when seeing the words on an ‘encouragement poster’, made by an inpatient. Martin describes this *more* in words such as ‘meeting the patients on their *healthy side*’ and as care focusing on having ‘*other* projects’ together than those related to the ED. He describes it as nursing care, that makes ill people momentarily forget their illness while participating in normal activities such as playing a game, playing piano, going for a walk, painting, knitting, or picking flowers. As Martin puts it, it is a nursing care focusing on the sick persons' ‘other wishes, other needs, other opportunities’.

The importance of nursing care, taking more than the patient's body and illness into consideration, is underlined by Nilsen et al. [9], de Vos et al. [8] and Rance et al. [10]. Nevertheless, whereas this research has pointed towards the necessity of nursing activities focusing on more than the body, it has seldom gone into depth about what this means and what it takes to meet patients with an ED ‘on their healthy side’. Based on the above lived experience description and the full conversation with Martin, this paper focuses on the three analytical questions: (1) *What does it mean to see* ‘more than the body’ *in nursing care for people with EDs? (2) What does it take—and how is it possible for nurses to open spaces where people with EDs also sense and take part in such ‘more’? (3) What kind of meaning and significance can such ‘more’ have for people with EDs and for the nurse offering care?* These questions were raised inductively in a dialogue between the two authors, while going through Martin's story.

#### 3.2.1 What Does It Mean to See ‘More Than the Body’ in Nursing Care for People With EDs?

Let us begin by particularly turning to what Martin sees and focuses on when he sees *more* than the patient's body.

Firstly, Martin tells us about seeing the *healthy side* of people. In the interview Martin tells us that these people often feel identified with the ED. In their family as well as in hospital, he describes how the ED often forms the whole picture, making it difficult to see or approach the person as something else than the disease. When Martin talks about seeing ‘more’ than the body and illness, he talks about situations where he gets glimpses of the person in front of him and dimensions of this person's life, *not* characterized by illness.

Secondly, when Martin talks about the healthy side, he describes it as an insight into an interest or activity that the patient usually has enjoyed. Martin further tells that often such interests have been forgotten for a longer or shorter time. Sometimes, Martins says, the patients have even been told to avoid their normal interests and activities because of their calorie burning effect. Seeing the healthy side is also described as a recognition of burgeoning hopes and dreams. The healthy side becomes apparent as, for example, an interest in music or a certain game. It can also show up as a former hobby—for example, playing the piano, or it can arise during an ordinary day at the ward in quite simple pleasures such as going for a walk or picking flowers. According to Martin, many such ordinary activities have been lost in the lives of people with EDs and they are not encouraged either at the ward for mainly two reasons. One is that the hospitalized patients often have been too ill to maintain ordinary activities. The other is that many such activities are restricted during hospitalization because of the need to reduce calorie burning. What Martin points out is that seemingly ordinary activities, which are often removed from the patient's lives, can offer a glimpse of being normal, healthy and of being something else than the ED.

Thirdly, Martin reflects on these activities as offering even more than normality. One thing is to get a sense of normality and get in contact with one's healthy side—and that is undoubtedly important for these people. Another is that inviting them to participate in activities connected to former interests or future dreams can be a threshold into the vibrancy of life. According to Martin, people with severe EDs find themselves in a very restrictive and rule‐bound world. In such a world, normal activities with no other purpose than having a pleasant moment form a contrast, offering a sense of meaning and a life worth living.

Seen through the lenses of philosophic aesthetics by Jørgensen [20, 21, 23], Martin's experience of patients who get a glimpse of something ‘more’ than their bodies and illness, can be described as an experience of intensified meaning and value arising during a normal rule‐bound and controlled day on the ward. When a person with an ED rediscovers the joy of playing the piano, it is in one way just a normal everyday activity. Nevertheless, according to Jørgensen [25], this playing of the piano could be much more than just everydayness. It can be an experience offering a sense of meaning and belonging to this world. In this way, normal activities can give rise to such aesthetic experiences as these, according to Jørgensen [21] characterized by a sense of the world opening up and even welcoming you. Following Jørgensen, Martin's stories about activities, that let him, and the patients see and sense something larger and more comprehensive than the patient's body and illness, can be understood as moments that open for other and larger dimensions in these peoples` lives. Martin shows that normal activities can offer opportunities to experience, that life holds more than body, more than weight, more than rules and restrictions. Life can be seen and experienced in another light and can be valuable and worth living. Jørgensen even goes so far as connecting such experiences of meaning to a possibility of coming ‘closer to an understanding of the human beings we are’ [21] p. 113.

The connection between a life worth living, a sense of oneself as a human being and glimpses of deep existential meaning in everyday activities in a psychiatric hospital ward points to the possible importance of such activities in the care of people with EDs.

#### 3.2.2 What Does It Take to Open Spaces in Which People With EDs Can Have a Sense of, and Take Part in ‘More’?

In the following, we will take a further step, asking: What does it require of the nurse to see ‘more than the body’ and to invite people with EDs to get a sense of this ‘more’?

In the interview with Martin, he talks about a lot of doors closing while being hospitalized with an ED. Some of the doors are real doors as many of the patients have restricted access to the outside world. Other doors are metaphorical doors, pointing to the fact that a lot of normal possibilities as well as hopes and dreams are closed for these patients.

Following Martin, we understand the small and normal activities as the crossing of a threshold between two kinds of spaces. Whereas the hospital ward can be understood as the large and overall room in which the patient stays for a few days, weeks or months, the smaller spaces of normal activities are of shorter duration, lasting only minutes or a few hours. Where the larger hospital ward is characterized by a focus on treatment by trying to control the illness, the minor spaces make the illness step back or even be forgotten for a short while. Moreover, where the large hospital room and its staff appeal to the patient to keep the disease on a short leash, the minor spaces appeal to the patient to loosen up, to forget themselves, and to give themselves over to activities which they enjoy.

Martin describes it like this:

We have treatment plans that are very detailed… And we probably get a little coloured by that, so maybe sometimes we should try to reduce the rules a little bit and then face them (the patients) a little bit more like life is out there, rather than meeting their rulesets with our rulesets. I think we can maintain the control and the coercion and the governance with that (Interview with Martin).

What Martin points to is also described by Rance et al. [10]: That too much focusing on rules and regulations around the ED can maintain the illness. This does not mean that restrictions and focus on the body and food are wrong or can be dispensed with. The point is that rules and regulations, and the focus on demarcation of the illness, cannot stand alone. An alternative to the illness is needed, and this alternative can very well be aesthetic experiences of meaning.

The question is, what does it take to lead the patient across the threshold into such minor and lively spaces? To that question, Martin offers two levels of reflection: one concerned with the patients, the other with the culture and values of the ward.

At the patient‐centered level, Martin tells us that it takes a lot of effort to encourage the patients to cross thresholds into activities that they earlier enjoyed. Often the patients have some reservations and often Martin's invitations are rejected at first. In the above lived experience description, Martin describes how he tries to push the patients empathetically. Martin further describes such empathetic pushing as finding a balance between respecting a ‘no’ from the patient at the same time maintaining and repeating the invitation. Other balances arise as some patients are ambivalent regarding their hopes and dreams for the future. According to Martin some patients hope to be freed of the ED, others hope to be able to continue their non‐eating practice. These balances between pushing empathetically on the one hand and accepting a no on the other, and between hoping to be well and hoping to continue being ill, call for constant professional and situationally anchored judgement. It is not at any time, invitations to activities are well placed.

At the level of the culture and values of the ward, it requires a lot from Martin to open a door into these minor spaces of joyful activities, as these spaces build on other logics that are most common in the culture of the ward. In the interview, Martin emphasizes a need to be courageous, as the minor spaces in many ways stand in contrast—almost opposition—to the rules and restrictions of the ward. Some of the rules Martin talks about concern restrictions regarding the patient's activity levels and food intake. Other rules are referred to as unwritten cultural rules. For example, Martin mentions a cultural unwritten and unspoken rule during the interview: that it is preferable, and expected of him as a nurse, to tidy up the kitchen, and that this seems to be considered more important than playing the piano or a game with a patient.

In that sense, these smaller spaces, which Martin finds so important, if people with an ED are supposed to recover, can be understood as a kind of ‘resistance space’ placed amid the larger cultural space of rules and regulations. It is important to underline that Martin does not talk about these smaller spaces as a possible alternative to the larger. Martin just points to the necessity of letting something else be present in addition.

Returning to the philosophy of Jørgensen [20, 21, 26]; aesthetic experiences, meaning experiences of the world opening and offering glimpses of intensified meaning, are experiences which we in the Western culture have been neglecting for a long time. Seen through the lens of Jørgensen [20, 21], Martin's description of a need to be courageous has reference to a long history of trusting logic and factual empirical knowledge more than what Jørgensen, with a reference to Baumgarten [24], describes as sensitive cognition.

When Baumgarten [24] founded aesthetic philosophy in the eighteenth century, it was by suggesting an alternative to the focus on cognition as solely intellectual and preoccupied with reason. “Baumgarten presented a new philosophy of human cognition, which involved an emotional kind of cognition, which he dubbed ‘sensitive cognition’” (20p.119). Jørgensen [20] further describes sensitive cognition as a cognition giving access to a sense of universal truths and a sense of greatness in life. The challenge here is that such cognition involves feeling, sensation, and presentiment—which differentiates it from logic and rational cognition.

Martins' description of the two logics in the ward represented by the larger space with a focus on rules and regulations trying to limit the illness and the smaller spaces of joyful activity trying to enlarge life very well illustrates Jørgensen's point [20, 21]: The western culture often forgets or neglects aesthetic experiences as important for us as human beings. The research of Nilsen et al. [9], de Vos et al. [8, 10] and Rance et al. [10] also demonstrates this with a call from the patients to enlarge the care focus by seeing more than their bodies.

Seen through the lens of Jørgensen [21] it is also possible to say that Martin needs different kinds of courage to open small spaces in which joy, meaning and liveliness can arise. Firstly, Martin needs courage to judge whether it is the right time and place for this specific patient to be involved in normal daily activities. Secondly, when Martin assesses that *now* is the right time and place for this specific patient, he needs courage to stand up against a long tradition in healthcare of placing greater confidence in intellectual reasoning than in experiences of meaning. What Martin must fight against then is a historically developed cultural confidence in the overall value of rules and restrictions around food‐intake and activity restriction as the main way to recovery for people with an ED.

#### 3.2.3 What Meaning and Significance Can ‘More’ Have for People With an ED and for the Nurse Offering Care?

The last question is, why is it important to invite people with an ED into these small spaces of liveliness and meaning?

In Martin's view, it is important for patients with an ED to visit these small spaces because they allow the patients to be taken away from themselves and the disease. Despite their severe illness, the patient has an opportunity to experience another world and a possible life in which the ED is not at the forefront. This other world gives the patient a break, but even more importantly, this other world offers hope. In these small spaces, the patient can get a feeling of a life containing much more than illness, food, calories, and restrictions. In these small spaces, it becomes possible for patients with an ED to have a sense that life could be otherwise. In this way, these spaces throw a little joy and a little ‘*light into the dark ED*’ (interview with Martin). According to Martin, this means that important doors which the disease has otherwise closed can be opened again.

In addition, these small spaces and their offering of participation in ordinary activities allow for a different contact between Martin and the patients. When Martin and a person with an ED play the piano or a game together, they meet and see each other in a different way through the game or through the music. According to the overall interview with Martin, this gives very nice contact and good times. These small spaces of normality and meaning are significant for the patients. For a short time, the patients are freed from their difficulties. It is also of significance for Martin to occasionally experience success in something he considers important: spreading a little light into a dark phase of the patient's life. Martin says:

You get to know these people well. And when you get a good relationship and a sense of trust, that's a very nice gift. In addition, when there is weight gain, and you notice that the patient becomes increasingly cognitively present, you notice that they want to realize themselves and take up interests. These are very small steps. It is rewarding to be able to help others in that way with such a special and complicated disorder (Interview with Martin).

In Jørgensen's aesthetic philosophy [20, 21], a sense of taking part in something larger plays a part in aesthetic experiences. Aesthetic experiences integrate ‘the individual into something bigger’ [20] p. 124. In aesthetic experiences, humans can get a brief experience of a cohesion far larger than we ever imagined, thereby also the possibility of actualizing ourselves in other and new ways. According to Jørgensen, such experiences, as underestimated they may be today, carry nothing less than a sense of ‘a truth of all that is’ [20] p. 123. Moreover, this truth tells us that something is of real value and that life can be worth living. It is also telling us that experiences of ‘being complete human beings’ [20] p. 133 are possible during a seemingly ruined life.

What Martin, in the light of Jørgensen [20, 21], shows us is that small, apparently unimportant, activities, such as playing a game, going for a walk, listening to music, or picking flowers are not at all unimportant. These activities can be seen as alternative spaces, where experiences of being whole and preoccupied with interests remind both patients and nurses of the existence of a ‘healthy side’. Moreover, these moments remind both patients and Martin that there is something valuable and worth living for. This is not lifesaving in a medical sense. Nonetheless, from an aesthetic philosophical perspective, these moments are certainly lifesaving, as they offer a sense of meaning, thereby adding a spark of hope to a life that has fallen apart.

In that sense, the above aesthetic philosophical reflections on Martin's lived experience description and interview respond to a call from patients recovering from EDs [8, 9, 10, 27], by showing how it is possible to let patients with EDs experience moments of meaning, thereby getting in contact with life as rich, and themselves as healthy.

## 4 Discussion

The research by de Vos et al. [8] on patients' perspectives on criteria for ED recovery states that health is not solely the absence of disease; it is also “the presence of something positive” [8] p. 2. This statement corresponds to WHO's declaration on mental health [28] as a state of mental well‐being that enables people to cope with the stresses of life, realize their abilities, learn well and work well, and contribute to their community.

This study shows that nursing care for people with severe EDs can—and should—include the presence of something positive in the everyday life of the patients. Joyful activities like playing piano or picking flowers can of course not stand alone in the care of this group of patients but are recommended as an important addition to the merely regulatory interventions.

The study shows that nurses caring for patients with severe ED must find a balance between two different scientific paradigms.

On the one hand, care must be undertaken in a space based on a rational paradigm and informed by scientific evidence leading treatment and care through a set of rules and regulations concerned with calorie‐counting, weight measurement, and restriction of activity level. In this space, the focus is on the disease and its regulation [2, 4, 5, 6, 7].

On the other hand, the care for patients with EDs must include invitations into other spaces based in another paradigm, where participation in everyday activities lets patients experience life as large, promising and meaningful. Care anchored in this last paradigm is here associated with Jørgensen's concept of aesthetic experiences and senses of deep existential meaning and belonging [22].

According to de Vos et al. [8], Nilsen et al. [9], Rance et al. [10, 27] much research within the field of EDs has been neglecting the importance of positive functioning for mental health. According to the nurse, Martin, professional practice also sometimes forgets the meaning and pleasure of being absorbed in an activity. This for sure is problematic. As Rance et al. [10] show, a one‐sided focus on the disease can encourage the ED conduct to continue.

The two presented paradigms can be understood in line with Antonovsky's differentiation between a pathogenetic and a salutogenetic approach [29, 30]. Whereas Antonovsky [29, 30] describes a pathogenetic approach as preoccupied with disease control and prevention, a salutogenetic approach focuses on conditions enhancing health. Antonovsky [30] shows that a one‐sided focus on curing diseases is too limited to understand how people are and become healthy. Instead Antonovsky [30] suggests focusing on salutary factors, meaning the factors in life which enhance the healthy side of human beings. He further shows that a sense of meaningfulness is a salutary factor adding significantly to health [30].

Although Antonovsky's theory is more than 30 years old, it still holds strong relevance and influence in the field of health promotion [31]. Antonovsky's [29, 30] theory supports the findings in this study, where seemingly ordinary activities such as playing a game or going for a walk can enhance a healthy side and a feeling of being ‘more than my body’. At the same time our findings show that salutogenetic and pathogenetic perspectives must be integrated in the care for patients with an ED.

This perspective raises questions on the balance between the different paradigms in nursing patients with severe ED. The question is how to balance a pathogenetic and a salutogenetic perspective or, put another way, the question is how to integrate disease control and health promotion in the nursing of patients with EDs?

Our findings show that such balances are difficult and require courage to break with norms and traditions of pathogenetic care for patients with EDs. Moving nursing of patients with an ED towards the patients' healthy side means to sometimes challenge the activity restrictions and calory counting—suggesting other focuses and shared activities. Our study further indicates that the integration of joyful activities in the care of people with severe ED relies on nurses and other healthcare staffs' continuous professional judgement. The balance depends on constant considerations regarding the right time and place to offer joyful activities to a specific patient. Such judgements cannot be rulebound processes but need to be carried out as constant situational anchored judgements. As Aristotle once wrote: «Nor is prudence (translates phronesis) a knowledge of general principles only; it must also take account of particular facts, since it is concerned with action, and action deals with particular things [32], p. 152. What Aristoteles once showed was that ethically good practice cannot rely solely on scientific knowledge and general principles. What is further needed is an account of particularity—meaning a sense of what is the right thing to do in this present situation. According to Aristoteles [32], the ability to judge is therefore needed, and this ability can only be developed through experience. Our study shows that nurses, in addition to scientific knowledge, must apply phronetic knowledge in the care of patients with a severe ED. This finding is confirmed in another study in the overall research project of EDs and care [13].

### 4.1 Strengths and Limitations of the Study

This study included empirical material from one nurse, and the patients' perspective is not included. This might be considered as both a weakness and a strength. Only one participant limits the possibility of transferability of the study, why a larger number of participants could have nuanced the findings and made it relevant in a larger context. At the same time, the limitation to one lived experience description and one phenomenological interview has given a possibility of doing a phenomenological in‐depth analysis which would not be possible with a larger empirical material.

The choice of Jørgensen's philosophic aesthetics as a theoretical deepening of the analysis is influenced by the first author's interest in care aesthetics. Such framing could of course be otherwise but added significantly to the understanding of how meaningful and important it is for human beings to participate in moments where life is experienced as large and promising.

## 5 Conclusion

This present study shows that nursing patients with severe ED must take ‘more than the body’ into consideration. Where earlier research and practice mainly have taken a pathogenetic approach, focusing on weight gain, calorie counting and activity restrictions, this study suggests adding salutary factors to the everyday life of care at the wards. Based on the findings, this study shows that taking care of ‘more than the body’ can be as simple as inviting patients with an ED into joyful activities like playing the piano, going for a walk, or picking flowers. Such activities might not seem as much, but from a philosophical aesthetic perspective, they can add a deep existential sense of meaning and belonging in this world—thereby adding to the patients’ healthy side.

Moreover, this study shows that a balance between pathogenetic and salutogenetic perspectives must be constantly searched for in the care for patients with an ED, why nurses in this field must apply phronetic knowledge.

On that basis, the study recommends a broader perspective on the care of patients with an ED in practice, including invitations into activities that bring with them a sense of life as large and promising.

This study recommends future research relying on larger data material, inquiring into the meaning of joyful activities for the healing and health of patients with a severe ED.

## Author Contributions

Study design: B.S.B., S.M.H.‐L., data collection: B.S.B., data analysis: S.M.H.‐L., B.S.B., manuscript preparation: S.M.H.‐L., B.S.B.

## Funding

The authors have nothing to report.

## Ethics Statement

The project, from which the interview data rise, is ethically approved by SIKT (The Norwegian data protection service) and conforms to the Declaration of Helsinki. This project did not receive any external funding.

## Conflicts of Interest

The authors declare no conflicts of interest.

## Data Availability

The data that support the findings of this study are available from the corresponding author upon reasonable request.
